# Diurnal-Rhythmic Relationships between Physiological Parameters and Photosynthesis- and Antioxidant-Enzyme Genes Expression in the Raphidophyte *Chattonella marina* Complex

**DOI:** 10.3390/antiox13070781

**Published:** 2024-06-27

**Authors:** Koki Mukai, Xuchun Qiu, Yuki Takai, Shinobu Yasuo, Yuji Oshima, Yohei Shimasaki

**Affiliations:** 1 Fisheries Technology Institute, Japan Fisheries Research and Education Agency, 122-7 Nunoura, Tamanoura-cho, Goto, Nagasaki 853-0508, Japan; 2 Institute of Environmental Health and Ecological Security, School of Environment and Safety Engineering, Jiangsu University, Zhenjiang 212013, China; xuchunqiu@ujs.edu.cn; 3 Laboratory of Marine Environmental Science, Faculty of Agriculture, Kyushu University, 744 Motooka, Nishi-ku, Fukuoka 819-0395, Japan; ytakai@agr.kyushu-u.ac.jp (Y.T.); oshima.yuji.493@m.kyushu-u.ac.jp (Y.O.); 4 Laboratory of Regulation in Metabolism and Behavior, Faculty of Agriculture, Kyushu University, 744 Motooka, Nishi-ku, Fukuoka 819-0395, Japan; syasuo@brs.kyushu-u.ac.jp

**Keywords:** diurnal rhythm, *Chattonella marina* complex, photosynthesis, antioxidant enzyme

## Abstract

Diurnal rhythms in physiological functions contribute to homeostasis in many organisms. Although relationships between molecular biology and diurnal rhythms have been well studied in model organisms like higher plants, those in harmful algal bloom species are poorly understood. Here we measured several physiological parameters and the expression patterns of photosynthesis-related and antioxidant-enzyme genes in the *Chattonella marina* complex to understand the biological meaning of diurnal rhythm. Under a light–dark cycle, Fv/Fm and expression of *psbA*, *psbD*, and *2-Cys prx* showed significant increases in the light and decreases during the dark. These rhythms remained even under continuous dark conditions. DCMU suppressed the induction of *psbA*, *psbD*, and *2-Cys prx* expression under both light regimes. Oxidative stress levels and H_2_O_2_ scavenging activities were relatively stable, and there was no significant correlation between H_2_O_2_ scavenging activities and antioxidant-enzyme gene expression. These results indicate that the *Chattonella marina* complex has developed mechanisms for efficient photosynthetic energy production in the light. Our results showed that this species has a diurnal rhythm and a biological clock. These phenomena are thought to contribute to the efficiency of physiological activities centered on photosynthesis and cell growth related to the diurnal vertical movement of this species.

## 1. Introduction

The raphidophyte *Chattonella marina* complex (hereafter referred to as *Chattonella*) is distributed widely in coastal areas of temperate and subtropical zones around the world and is known as one of the harmful algal bloom (HAB) species that cause mass mortalities of aquacultured fish around the world [1,2,3,4]. This species can form dense blooms on the water surface in midsummer during periods of high temperatures and light conditions. Generally, photosynthetic organisms generate higher amounts of reactive oxygen species (ROS) such as superoxide anion (O_2_^•−^), hydrogen peroxide (H_2_O_2_), and hydroxyl radicals (^•^OH) through photosynthesis when they are exposed to high temperatures and strong light conditions [5,6]. Because these ROS attack various cellular molecules, such as DNA, RNA, proteins, and lipids, high amounts and long-term exposure to ROS have serious adverse effects on living organisms [7,8]. Previously, it has been reported that D1 protein—an essential subunit of photosystem II—is decomposed by excessive ROS generated by strong light energy, leading to a decrease in photosynthetic activity [9,10,11,12]. In addition, *Chattonella* is known to generate higher amounts of ROS compared to other unicellular algae, including HAB species [13,14], and high concentrations of ROS are considered one of the factors that damage the gills of fish during red tide events [15,16]. Hence, this source of excessive oxidative stress produced as a byproduct of photosynthesis must be quickly removed from the cell.

Antioxidant enzymes serve an important function by regulating oxidative stress to maintain intracellular homeostasis. Photosynthetic organisms can also protect themselves from oxidative stress by decomposing ROS using antioxidants and antioxidant-enzyme systems [17,18]. In our previous study, the expression levels of antioxidant-enzyme genes in *Chattonella* increased with the increase in oxidative stress due to excessive light energy or H_2_O_2_ treatment [19,20], suggesting that the function of the antioxidant enzyme was to respond in a way that protected the cells from excessive oxidative stress. In general, photosynthesis has diurnal characteristics related to the movement of the sun; thus, the expression of antioxidant enzymes is also thought to have a similar rhythm for efficient removal of ROS and to be controlled by the biological clock.

Diurnal rhythms are defined as biological rhythms that, under given circumstances, complete a cycle in less than 24 h, are endogenously generated, and are continuous [21]. Diurnal rhythms have been studied in a variety of organisms, including photosynthetic organisms [22,23,24,25,26,27]. These rhythms contribute to the regulation of physiological rhythms within an individual and to efficient energy production, metabolism, growth, and behavior. Diurnal rhythms are closely related to photosynthetic activity. Kloppstech [28] was the first to report that in higher plants (the pea *Pisum sativum*), transcription of the genes for the light-harvesting complex, ribulose 1,5-bisphosphate carboxylase/oxygenase, and early light-induced protein follows a diurnal rhythm. These gene transcriptions showed a tendency to increase during the day and decrease at night. Moreover, the expression of *psbA* and *psbD* genes, which code for the D1 and D2 proteins of photosystem II, follows a diurnal rhythm in higher plants and cyanobacteria [29,30,31,32].

Most phytoplankton with flagella, including *Chattonella*, are known to show diurnal vertical migration (DVM) in the ocean. The biological significance of DVM is thought to be for sufficient photosynthesis in the surface layer during the daytime and for nutrient supplementation in the deeper layers at night [33]. DVM may also play a role in mitigating photosystem damage by adjusting the water depth occupied. DVM in some flagellate algae has been suggested to follow a diurnal rhythm controlled by an endogenous clock [34,35]. Shikata et al. [36] reported that in the raphidophyte *Chattonella*, the diurnal rhythm in DVM is regulated by blue light. They concluded that *Chattonella* can sense the weak blue wavelength from sunlight throughout its depth range, allowing it to cue its DVM to the day–night cycle regardless of weather and transparency. In addition, they also reported that this species changes its taxis in response to different wavelengths of light [37]. In natural environments, the wavelengths of light received vary depending on the weather and time of day, and it is thought that they act based on this information. Also, in our previous study, we found significantly higher levels of the *Chattonella* proteins OEE, Cyt c553, and AtpB during the light period, whereas GAPDH and RPL12 generally showed higher expression levels during the dark period [38]. These diurnal biological responses are thus considered essential for efficient energy production using light. Studying the diurnal rhythm in *Chattonella*, which responds sensitively to light conditions, is expected to provide not only an understanding of its ecology but also knowledge that will help mitigate economic losses from red tides involving this species. However, any relationships between diurnal rhythms and gene expression, photosynthetic activity, and oxidative stress are as yet unclear in HAB species, including *Chattonella*.

*Chattonella* in particular is expected to have a high degree of oxidative stress tolerance related to the diurnal rhythm due to its ecological characteristics of forming blooms under conditions of high water temperature and high light intensity during the summer in Western Japan. It is therefore expected that *Chattonella* will be a model organism for studying oxidative stress tolerance in marine phytoplankton. Here, we analyzed the diurnal patterns in the expression of photosynthesis-related genes (*psbA* and *psbD*) and those for antioxidant enzymes in *Chattonella*. Moreover, we investigated diurnal photosynthetic activity (Fv/Fm ratio), oxidative stress level (O_2_^•−^ and H_2_O_2_ production), and H_2_O_2_ scavenging activity, and we analyzed their relationships with the expression of these genes to understand the biological significance of their diurnal patterns.

## 2. Materials and Methods

### 2.1. Experimental Design

An axenic strain of *Chattonella marina* var. *antiqua* (Hada) Demura & Kawachi (NIES-1) was obtained from the National Institute for Environmental Studies (Tsukuba, Ibaraki, Japan). This strain was grown at 25 °C under a 14 h light:10 h dark (14L:10D) photoperiod at 100 μmol photons m^−2^ s^−1^ supplied by LED bulbs (Toshiba, Tokyo, Japan) using modified SWM3 medium at a salinity of 30 [39]. The culture medium was shaken once a day.

**Figure 1:**
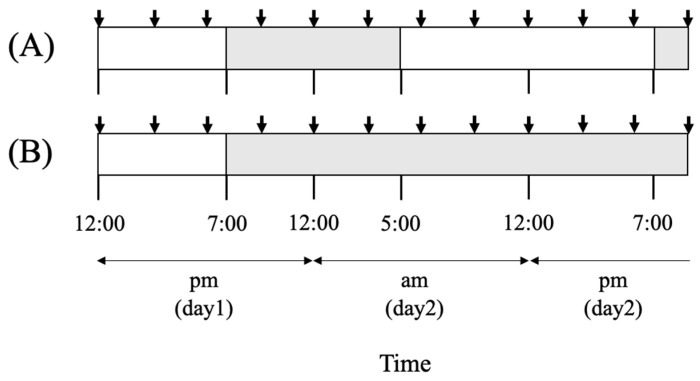
Diagram showing the sampling times of cultures of *Chattonella* under light conditions of 14 h light:10 h dark (LD) (**A**) and continuous dark (24D) (**B**) after initial 7 h irradiation. Samples were collected at 12 time points throughout the experiment as indicated by arrows: at 12:00 (noon), 3:00, 6:00, and 9:00 p.m. on day 1, and at 12:00 (midnight), 3:00, 6:00, and 9:00 a.m. and 12:00 (noon), and 3:00, 6:00, and 9:00 p.m. on day 2. The white background shows periods of irradiation, and the gray background indicates the dark periods.

A *Chattonella* cell suspension in early stationary phase (10,550 cells mL^−1^, 3 L) was used for the diurnal rhythm experiment. About 2 L of this suspension was dispensed into eight flasks (240 mL each, Thermo Fisher Scientific, Tokyo, Japan) and cultured for 24 h under the same light and temperature conditions as the preculture to recover from any stress from dividing the culture. At 12:00 a.m. of the following day, four flasks were placed under each of two light regimes: 14L:10D (hereafter “LD”) and continuous dark (“24D”). All flasks were cultured for 33 h, including an initial 7 h light irradiation period (Figure 1). Samples were collected from 4 flasks of each group (*n* = 4) every 3 h (13.5 mL from one flask). Soon after sampling, a portion of each cell suspension sample was used for cell counts using microscopy at 40× magnification, Fv/Fm measurement, O_2_^•−^ and H_2_O_2_ detection, and measurement of H_2_O_2_ scavenging activity. The remaining portion of the cell suspension was centrifuged (1800× *g* for 10 min), and the cell pellet was stored at −80 °C until RNA extraction for determining the expression of photosynthesis-related and antioxidant-enzyme genes using quantitative PCR (qPCR).

### 2.2. Measurement of the Fv/Fm Ratio

Fv/Fm, which indicates the maximum quantum yield of photosystem II, was measured using an Aqua Pen fluorometer (PSI, Photon Systems Instruments, Czech Republic) as described in our previous study [40]. To investigate the effect of different photoperiods on photosynthetic performance, we used 2 mL of each cell suspension sample. Cell suspensions were kept in the dark for 30 min before measurements.

### 2.3. Detection of O_2_^•−^ and H_2_O_2_ and Measurement of H_2_O_2_ Scavenging Activity

O_2_^•−^ was measured in accordance with the methods of Kim et al. [41]. First, 5 μL of modified SWM3 medium or superoxide dismutase (SOD; 1000 U/mL) was mixed with 145 μL of a cell suspension in a test tube. Next, 50 μL of 10 μM 2-methyl-6-(p-methoxyphenyl)-3,7-dihydroimidazo [1,2-]pyrazin-3-one (MCLA) was added. The chemiluminescence response was then recorded immediately for 20 s at room temperature using a tube luminometer (Lumat LB 9507; Berthold Technologies, Tokyo, Japan). The chemiluminescence intensity for each sample was corrected by subtracting the measurement with added SOD (SOD+) from the reading without added SOD (SOD−).

H_2_O_2_ concentrations and scavenging activities were measured as in our previous study [19]. Briefly, the H_2_O_2_ concentration was measured by the luminol reaction-based method using a tube luminometer (Lumat LB 9507; Berthold Technologies) and calculated using a standard curve of H_2_O_2_ concentrations from 0 to 0.1 mM. We measured H_2_O_2_ scavenging activity as catalase activity equivalents using a tube luminometer (Lumat LB 9507; Berthold Technologies). We then calculated H_2_O_2_ scavenging activity based on the decrease in chemiluminescence of standard samples (0, 10, 20, 30, 40, and 50 units of catalase in 50 μL medium).

### 2.4. Gene Expression Analysis

Expression of the photosynthesis-related and antioxidant-enzyme genes was analyzed by qPCR following our previous studies [20,42]. Briefly, total RNA was extracted from the cell pellet (see Section 2.1) using an ISOSPIN Plant RNA (Nippon Gene Co., Ltd., Tokyo, Japan). The extracted total RNA was treated with DNase I, which was included with the commercial kit. Total RNA was quantified using a spectrophotometer (BioSpec-nano; Shimadzu, Kyoto, Japan). cDNA was synthesized from 400 ng of total RNA using Primer Mix, which included a random primer and an oligo (dT) primer in the ReverTra Ace^®^ qPCR RT Kit (Toyobo, Osaka, Japan). The expression level of each photosynthesis-related and antioxidant-enzyme gene was measured using a Mx3000P Multiplex QPCR System (Stratagene, Tokyo, Japan).

The primers and probes used in this study are shown in Table 1. Original primers for photosynthesis-related genes were designed using sequences that had been previously deposited in the DNA Data Bank of Japan (DDBJ) (*psbA*, LC595635; *psbD*, LC595637). The expression of photosynthesis-related genes was then analyzed using the THUNDERBIRD SYBR qPCR Mix (Toyobo) following the manufacturer’s protocol. The expression of antioxidant-enzyme genes was also analyzed following our previous study [20]. We selected calmodulin (*CAL*) as a reference gene as it showed the most stable expression levels among commonly used reference genes (*cytochrome c oxidase subunit 2* and *elongation factor*). We then used the RefFinder tool [43], which integrates four specific algorithms (GeNorm by Vandesompele et al. [44], NormFinder by Andersen et al. [45], BestKeeper by Pfaffl et al. [46], and the comparative delta-Ct method by Silver et al. [47]) to assess and screen these candidate reference genes. The reaction mixture and thermal conditions are described elsewhere [20,42].

### 2.5. Regulation of psbA, psbD, and 2-Cys prx Gene Expression by the Electron Transport Chain in Photosystem II

The expression of some genes encoded in the chloroplast genome is known to be regulated by the redox state in the plastoquinone pool within the photosystem [48,49,50,51,52]. We investigated whether the expression of *psbA*, *psbD,* and *2-Cys prx*, which are expected to be encoded in the chloroplast genome, is regulated by the redox state in the plastoquinone pool by using 3-(3,4-dichlorophenyl)-1,1-dimethylurea (DCMU), which blocks the electron transport at quinone B in photosystem II.

A culture of *Chattonella* grown under the conditions described in Section 2.1 until the early stationary phase (24,466 cells mL^−1^) was divided among 16 flasks (100 mL each) at 4:00 p.m. and maintained under the same conditions until midnight. DCMU (dissolved in ethanol) was then added to 8 of the flasks (final DCMU concentration, 10 μM; final ethanol concentration, 0.01%), and ethanol only was added to the other 8 flasks as a solvent control. For this experiment, four flasks were used in each treatment: DCMU + light, DCMU + dark, solvent control + light, and solvent control + dark. For the treatments with light, irradiation started at 5:00 a.m., whereas dark treatments were continuously kept in the dark. These groups were sampled (10 mL from 1 flask in each treatment) every 3 h until 9:00 a.m. (midnight, 3:00, 6:00, and 9:00 a.m.). Analysis for expression of *psbA*, *psbD*, and *2-Cys prx* was performed using qPCR following the method described in Section 2.4.

### 2.6. Statistical Analysis

We used CircWave 1.4 software [53] (http://www.rug.nl/fwn/onderzoek/programmas/biologie/chronobiologie/downloads/index; accessed on 4 February 2005) to test for significant rhythmicity of Fv/Fm, O_2_^•−^ and H_2_O_2_ concentrations, H_2_O_2_ scavenging activity, and expression of photosynthesis-related and antioxidant-enzyme genes. CircWave 1.4 can be seen as an extension of cosinor analysis, and it uses a linear harmonic regression fit that describes the data by adding harmonics to the principal wave function. We adopted a significance level in rhythmicity of <0.01 to reduce the chance of false positives. An *r*^2^ > 0.05 was judged as a significant cosinor fit following CircWave analysis.

Relationships between expression levels of *psbA* and *psbD*, Fv/Fm, and O_2_^•−^ and H_2_O_2_ concentrations were analyzed using Pearson’s correlation coefficient and Statcel 3 (OMS, Saitama, Japan) in Excel. We also analyzed the relationships between expression levels of antioxidant genes and Fv/Fm, O_2_^•−^ and H_2_O_2_ concentrations, and H_2_O_2_ scavenging activity. These analyses were carried out using data from 6:00 a.m. to 9:00 p.m. on day 2.

In DCMU treatment, Student’s *t*-tests were used to detect significant differences in each treatment group.

## 3. Results

### 3.1. Cell Densities and Fv/Fm Ratios under Different Light Regimes

**Figure 2:**
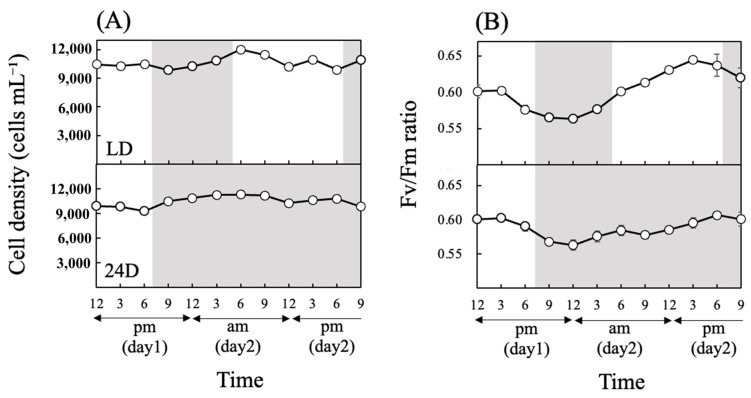
Cell densities (**A**) and Fv/Fm ratios (**B**) in cultures of *Chattonella* under two light regimes: 14 h light:10 h dark (LD), and continuous dark (24D). The top half of each panel shows LD conditions, and the bottom half is 24D. A white background indicates a light period, and a gray background indicates a dark period. The light period under LD was from 5:00 a.m. until 7:00 p.m. For 24D, the dark period started at 7:00 p.m. Values are mean ± SD (*n* = 4).

During the experiments, *Chattonella* cell densities ranged from 9900 to 12,000 cells/mL under LD and from 9250 to 11,300 cells/mL under 24D (Figure 2A). Fv/Fm reached a maximum during the light period and a minimum during the dark period under LD (Figure 2B). Fv/Fm under 24D showed a pattern similar to that under LD, but the maximum value was lower than under LD. CircWave analysis found diurnal rhythmicity in Fv/Fm under both light regimes, but the rhythmicity was higher under LD than 24D (Table 2).

### 3.2. O_2_^•−^ and H_2_O_2_ Concentrations and H_2_O_2_ Scavenging Activity under Different Light Regimes

**Figure 3:**
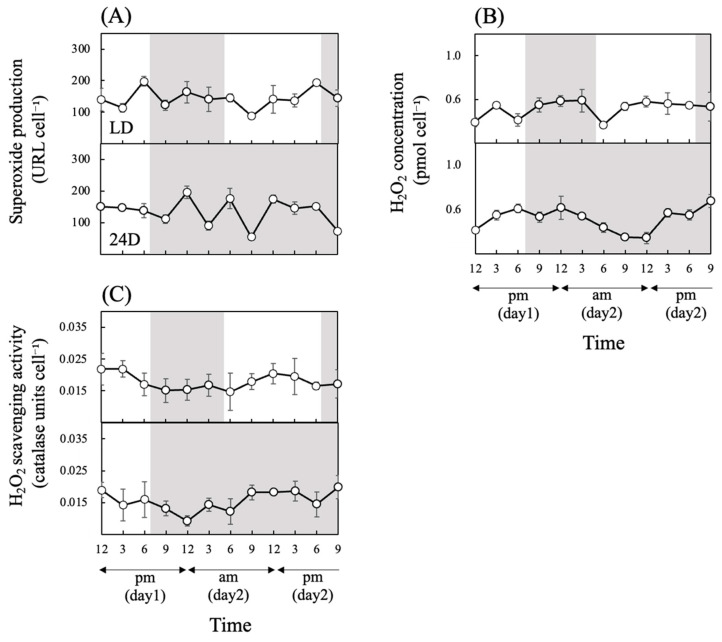
Superoxide production (**A**), H_2_O_2_ concentrations (**B**), and H_2_O_2_ scavenging activity (**C**) in cultures of *Chattonella* under different light regimes. H_2_O_2_ scavenging activity is expressed in units of catalase activity (units/cell). A white background indicates a light period, and a gray background indicates a dark period. The light period under LD was from 5:00 a.m. to 7:00 p.m. For 24D, the continuous dark period started at 7:00 p.m. Values are mean ± SD (*n* = 4).

There was no clear diurnal rhythmicity in O_2_^•−^ production under LD or 24D treatments (Figure 3A and Table 2) or in H_2_O_2_ concentration under LD (Figure 3B and Table 2). CircWave analysis detected significant rhythmicity in H_2_O_2_ concentration under 24D; however, due to the extremely low acrophase, this does not indicate a diurnal rhythm caused by photoperiod.

In contrast, H_2_O_2_ scavenging activity appears to be more prone to diurnal rhythms than O_2_^•−^ production and H_2_O_2_ concentration (Figure 3C). In particular, the rhythmicity of the activity under 24D is significant (Table 2), suggesting higher H_2_O_2_ scavenging activities during light periods controlled by the biological clock.

### 3.3. Expression Levels of Photosynthesis-Related and Antioxidant-Enzyme Genes under Different Light Regimes

**Figure 4:**
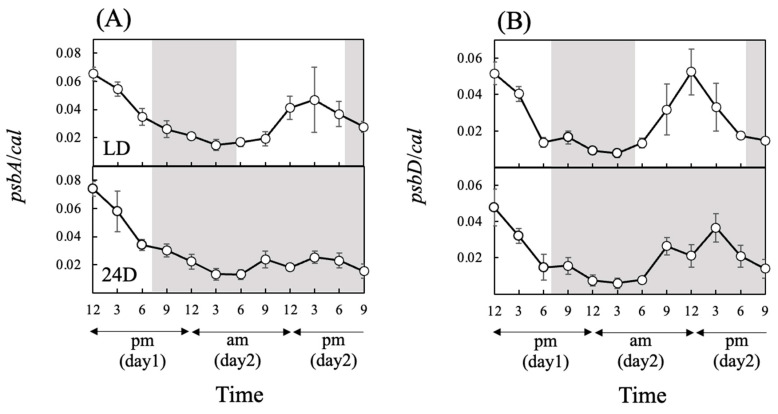
Relative gene expression levels of *psbA* (**A**) and *psbD* (**B**) in cultures of *Chattonella* under different light regimes, as determined by qPCR analysis. White background shows light periods, and gray background shows dark periods. The light period under LD was from 5:00 a.m. to 7:00 p.m. For 24D, the continuous dark period started at 7:00 p.m. Values are mean ± SD (*n* = 4).

Expression levels of both *psbA* and *psbD* under LD were higher during the light period than in the dark during day 2 (Figure 4A,B) and showed significant rhythmicity (Table 2). This rhythmicity was also observed under 24D, suggesting regulation by the biological clock.

**Figure 5:**
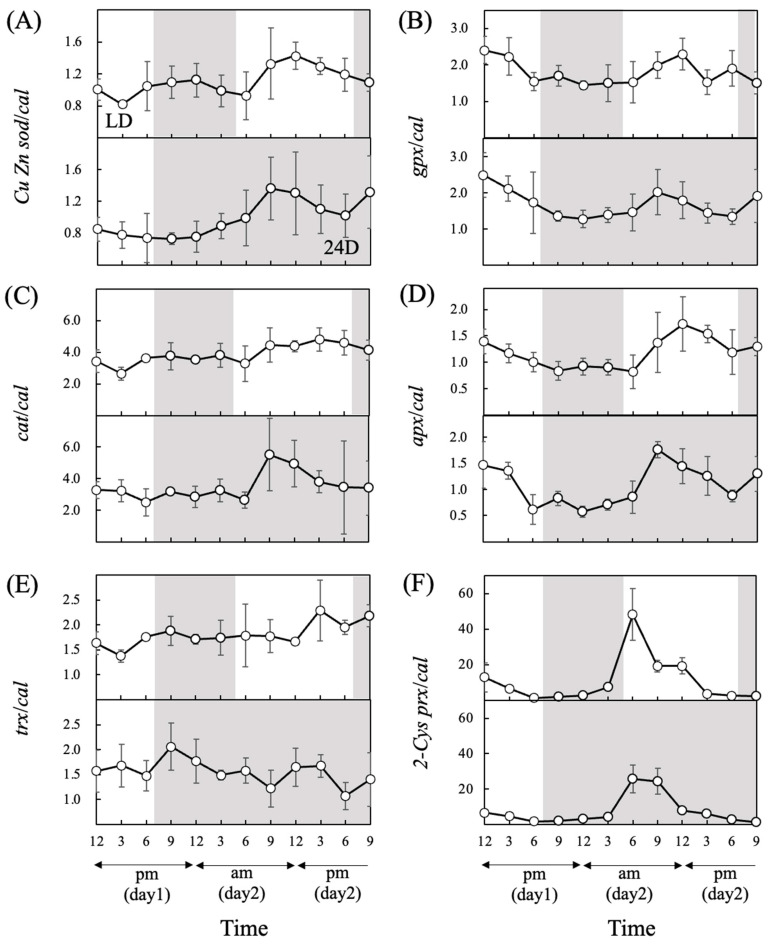
Relative gene expression levels of *Cu/Zn sod* (**A**), *gpx* (**B**), *cat* (**C**), *apx* (**D**), *trx* (**E**), and *2-Cys prx* (**F**) in cultures of *Chattonella* under different light regimes, as determined by qPCR analysis. A white background indicates light periods, and a gray background indicates dark periods. The light period under LD was from 5:00 a.m. to 7:00 p.m. For 24D, the continuous dark period started at 7:00 p.m. Values are mean ± SD (*n* = 4).

The expression of almost all antioxidant-enzyme genes under both light regimes showed diurnal rhythmicity, except for *gpx* and *trx* under LD and *trx* under 24D (Figure 5 and Table 2). The expression of *2-Cys prx* showed the fastest increase among these genes under both light regimes at 6:00 a.m. (Figure 5F).

### 3.4. Relationship Analysis Using Pearson’s Correlation Coefficients

The *psbA* expression and Fv/Fm under LD were significantly co-related, but not under 24D (Table 3). The expression of *psbD* was negatively related to H_2_O_2_ production under 24D. The expression of *apx* was significantly and negatively co-related with Fv/Fm and O_2_^•−^ production under 24D. *2-Cys prx* expression and Fv/Fm were significantly co-related, and H_2_O_2_ production was negatively co-related under both LD.

### 3.5. Suppression of psbA, psbD, and 2-Cys prx Expression by DCMU Treatment

**Figure 6:**
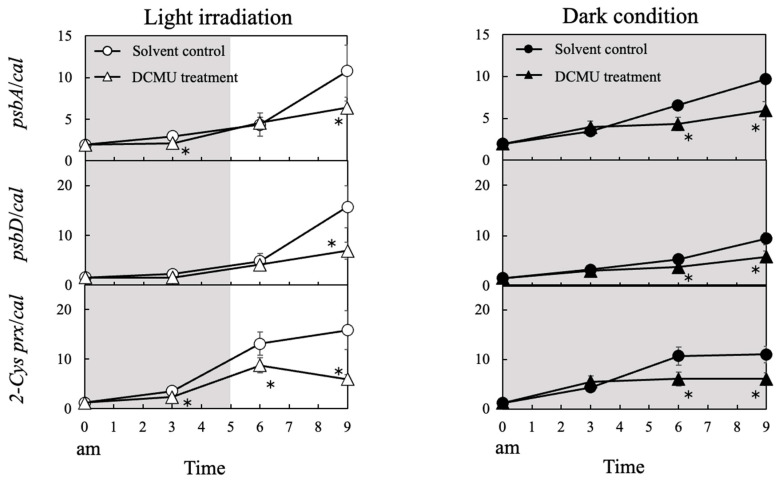
Gene expression response of *psbA*, *psbD*, and *2-Cys prx* in *Chattonella* treated with DCMU. A white background indicates light periods, and a gray background indicates dark periods. The left and right panels represent LD and 24D conditions, respectively. White circles, solvent control + light conditions; white triangles, DCMU + light; black circles, solvent control + dark; black triangles, DCMU + dark. DCMU, or ethanol (as a solvent control), was added at midnight. Values are mean ± SD (*n* = 4). * *p* < 0.05 (Student’s *t*-test was used to compare solvent control and DCMU treatment groups under LD and 24D conditions).

We investigated whether the expression of *psbA*, *psbD*, and *2-Cys prx*, which are expected to be encoded in the chloroplast genome, is regulated by the redox state in the plastoquinone pool. We used DCMU, which blocks the electron transport at quinone B in photosystem II. After irradiation with light, the increase in the expression of *psbA*, *psbD,* and *2-Cys prx* was significantly suppressed by DCMU treatment under both light and dark conditions (Figure 6).

## 4. Discussion

This study revealed the diurnal rhythm of some physiological parameters in *Chattonella,* such as photosynthetic activity and the expression of photosynthesis-related and antioxidant-related genes under LD conditions. The rhythm of many parameters was maintained even under 24D, indicating the existence of a biological clock in this species. We consider these findings to have an important role in maintaining the suitable physiological condition of *Chattonella*. Fv/Fm, a commonly used indicator for photosystem II activity, showed an increasing trend during the light period and a decreasing trend during the dark period under LD (Figure 2). The rhythmicity of Fv/Fm under 24D was weaker than that under LD, but a significant rhythm remained, with acrophase similar to that under LD (Table 2). These results strongly suggest that a variety of photosynthesis-related proteins have expression patterns that match the day–night rhythm.

In *Porphyra umbilicalis*, Fv/Fm was higher during the light period [54], but it increased in the dark in an endosymbiotic alga, *Symbiodium* [55]. These results suggest species-specific or habitat-dependent variation in the diurnal rhythm of photosynthetic activity. In another strain of *Chattonella*, no diurnal rhythmicity was observed in Fv/Fm [56]. However, in this study, *Chattonella* was cultured at a different light intensity and studied at a different growth stage than in the present study. These differences might affect the rhythms and patterns of gene and protein expression, and future studies should investigate changes in the rhythms in such cases.

*psbA* and *psbD* are genes encoding the D1 and D2 proteins, respectively, which are essential subunits for photosystem II [57,58]. In our experiments, the expression of *psbA* and *psbD* also showed significant diurnal rhythm under LD and 24D (Table 2). The significant positive correlation between *psbA* expression and Fv/Fm was detected under LD but not under 24D (Table 3). In general, the D1 protein is susceptible to photodegradation. We presume that the D1 protein was decomposed by the light energy at a fixed rate, even at the light intensity of this study, because the light intensity (100 μmol photons m^−2^ s^−1^) is near the saturation intensity for growth [59]. Thus, degradation and renaturation of the D1 protein were thought to affect Fv/Fm and the level of *psbA* expression, and a significant positive correlation between *psbA* expression and Fv/Fm might be detected under LD but not under 24D. Previous studies reported that the expression of *psbA* and *psb*D increased immediately after the start of light irradiation to activate photosynthesis in some photosynthetic species [30,60,61]. Hence, the patterns of *psbA* and *psbD* expression in the present study show that the photosynthetic activity of *Chattonella* is strongly supported by the D1 and D2 proteins.

Antioxidant systems are necessary for protection from harmful oxidative stress generated via biological processes. For example, photosynthetic organisms with mutants of antioxidant enzymes like 2-Cys Prx and Apx showed lower growth rates and photosynthesis activity with higher stress sensitivity [62,63]. In the present study, the expression of 4 and 5 out of 6 antioxidant-enzyme genes showed a significant diurnal rhythm under LD and 24D, respectively (Table 2). These results indicate that the expression of genes for these antioxidant enzymes is regulated by the biological clock to prepare for efficient ROS removal.

All of the antioxidant enzymes analyzed in this study, except for Cu/Zn SOD, are involved in peroxidase activity. H_2_O_2_ scavenging activity can be regarded as the sum of these multiple peroxidase activities. This activity exhibited a significant diurnal rhythm under 24D, and the activity under LD also showed fluctuations resembling a diurnal rhythm, although these were not significant. On the other hand, the expression of these peroxidase-related enzymes had no correlation with H_2_O_2_ scavenging activity (Table 3). In *Chattonella*, 2-Cys Prx is a major hydrogen peroxidase that accounted for 4% of all the protein spots visualized by fluorescent staining in the 2-DE profile [64]. Qiu et al. [38] reported that the expression levels of 2-Cys Prx protein did not change throughout a 12 h light:12 h dark diurnal cycle with 220 μmol photons m^−2^ s^−1^ of light intensity during the light period. In addition, we reported previously that the expression of *Chattonella 2-Cys prx* and H_2_O_2_ scavenging activity was induced by an increase in oxidative stress resulting from strong light irradiation at 1000 μmol photons m^−2^ s^−1^ [19]. Therefore, the 2-Cys Prx protein level may be regulated by its translation level, and the translation activity may be upregulated by an illuminance of at least 1000 μmol photons m^−2^s^−1^ but not by 100–220 μmol photons m^−2^ s^−1^. The expression level of other antioxidant-enzyme genes may not always be directly linked to the protein expression level under the light level in this study, and these factors may affect the relationship between the expression of antioxidant enzyme genes and H_2_O_2_ scavenging activity.

Under LD, the expression of *psbA*, *psbD,* and *2-Cys prx*, which are expected to be encoded in the chloroplast genome, was strongly induced during the light period. These gene expression levels were higher than those under 24D. In addition, the expression of all antioxidant-enzyme genes expected to be encoded in the nuclear genome, except for *gpx* and *trx*, also showed significant diurnal rhythm under the light period of LD. These results indicate the involvement of light energy in gene expression. The redox state in the plastoquinone pool in the electron transport chain within the photosystem is known to regulate the expression of *psbA* and *psbD* [48,49,50,51,52]. In addition, the expression of many photosynthesis-related genes encoded in the nuclear genome is known to be regulated by the plastid-to-nucleus retrograde signal pathway [65], by which the redox state of the photosynthetic electron transport chain, trans-thylakoid potential. This pathway is thought to control the gene expression rhythm in this study.

We also detected a significant diurnal rhythm in all genes except for *trx* under 24D. To investigate the mechanism of gene induction under dark conditions, we carried out an experiment using DCMU, which blocks electron transport within photosystem II. The induction of *psbA*, *psbD*, and *2-Cys prx* expression was suppressed after DCMU treatment compared with the solvent control group in both light and dark conditions. This result indicated the existence of a mechanism of gene induction by the redox state in the photosystem. In addition, these suggest the enhancement of the redox state of the photosynthetic electron transport chain under dark conditions by factors other than light energy, such as the provision of reducing power from the mitochondrial respiratory chain. If this speculation is correct, it is possible that the biological clock-like rhythms found in the expression of chloroplast genes in *Chattonella* are regulated by factors like a mitochondrial respiration chain. In higher plants, a protein highly homologous with the NAD(P)H dehydrogenase (NDH) complex in the mitochondrial respiratory chain was discovered in the chloroplast genome [66,67] and has been revealed to mediate electron transport on the thylakoid membranes. NDH is also known to transfer electrons to plastoquinone in higher plants. However, the NDH gene has not yet been found in the chloroplast genome of algae, so there may be a different electron transport pathway under dark conditions in *Chattonella* than in higher plants. Further research is needed on the diurnal rhythm of algal chloroplast gene expression.

**Figure 7:**
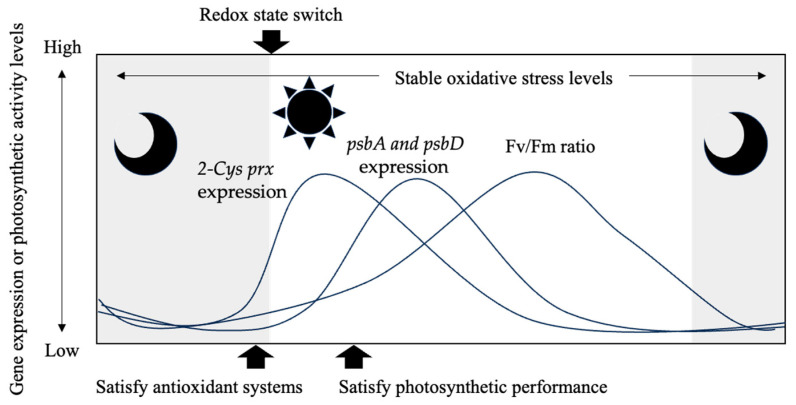
Summary of results obtained from this study.

Many studies have revealed that flagellated harmful algae start to swim toward the surface layer before dawn and swim to deeper layers toward night, e.g., [68,69,70]. Shikata et al. [37,71] reported that *Chattonella* begins to swim to the upper layer before light irradiation begins in its DVM behavior. Therefore, the initiation of DVM is expected to be controlled by the biological clock as well as light irradiation, especially blue light. In addition, they also reported that this species possesses the cryptochrome gene, which is sensitive to blue light [72]. Hence, it is possible that the behavior and physiological rhythms of this species are regulated through these photoreceptors. However, the underlying molecular mechanism of *Chattonella* behavior that starts before light irradiation, as well as the taxis (e.g., phototaxis or geotaxis), are poorly understood. The downstream cascade from regulation by clock genes is expected to be complex. In the present study, *2-Cys prx* expression increased before light irradiation, as in the DVM rhythm. Thus, in the environmental populations of this species, it is possible that vertical migration to the surface layer begins before receiving light to satisfy antioxidant systems (Figure 7). This is likely an important physiological rhythm in preparation for active photosynthesis. Also, in this study, a single species was cultured, but in a natural environment, competition with other species may occur, which may affect the physiological rhythms and may result in disruption of the diurnal rhythm of this species [73]. Further study is needed to reveal the molecular mechanism and ecological significance of the diurnal rhythm in this species.

## 5. Conclusions

This study is the first to report the relationship between several physiological parameters and the expression patterns of photosynthesis-related and antioxidant-enzyme genes in the *Chattonella marina* complex based on diurnal rhythm. We revealed that Fv/Fm, an indicator of photosynthetic activity, showed significant diurnal rhythm with *psbA* and *psbD*, a photosynthesis-related gene, and *2-Cys prx*, an antioxidant-related gene, under a light–dark cycle. Interestingly, these rhythms remained even under continuous dark conditions. These results suggest that this system has developed to enable efficient photosynthesis and protect itself from the oxidative stress that occurs. On the other hand, DCMU suppressed the induction of *psbA*, *psbD*, and *2-Cys prx* expression under both light regimes, but not completely. The existence of a gene expression mechanism independent of electron transport in the photosystem was predicted. Many molecular mechanisms of physiology and gene expression remain unknown in HAB species. Further studies are also needed to understand their diurnal-based physiological and field growth characteristics.

## Figures and Tables

**Table 1:** Primer sets used in this study.

Gene		Sequence (5′–3′)	Amplicon Size (bp)	Accession No.	Reference
*psbA*	Forward (F)	GAGCGCTTCTGCTCTTGGATTACT	143	LC595635	Original
	Reverse (R)	CCATCGATGTCTACTGGAGGAGCA			
*psbD*	F	CTTCGACCTTGTAGACGATTGGT	211	LC595637	
	R	ACTGTTCGCTGGTGTTGAAACAG			
*Cu/Zn sod*	F	GGACCGATTGGTGAAGCTCATAGG	162	LC337662	[20]
	R	GAGGATGCAATCCCAACAACTCCTC			
	Probe (P)	56-FAM/GTGGACATG/ZEN/AACTTTC GCTGCT/3IABkFQ			
*gpx*	F	GGTGAGTGGCTTCACGTACAATC	224	LC337663	
	R	CCGAAGCAACGTTAACTACAAGG			
	P	56-FAM/TGGAAACAT/ZEN/GAAAG GCCTTCTCGCAC/3IABkFQ			
*cat*	F	GCACATTTTGATCGTGAGCGTATCC	224	LC337664	
	R	TTCACTGCAAACCCACGAGGATC			
	P	56-FAM/GGGTACTTT/ZEN/GAGGTCA CAACTCTC/3IABkFQ			
*apx*	F	GCACTGACATGCCACAAGAGAAATG	208	LC337665	
	R	TCAAAGACCAATGGCTCTTGAGTCC			
	P	56-FAM/ACATCTTTG/ZEN/GCCGCATGGAAT/3IABkFQ			
*trx*	F	TCTCCGATCTCCGTGTTGATTTTGC	145	LC337666	
	R	GGTTTCCTGATTGCCTTTTGCGC			
	P	56-FAM/ACCAAAGGT/ZEN/GTACAGGAATCCTC/3IABkFQ			
*2-Cys prx*	F	TCAAGAAAACCCGGATGAGG	139	LC337661	[19]
	R	GGCATAATCTTAGAAACGAG			
	P	56-FAM/AGCCAGATC/ZEN/CTGTC GGCTCT			
*Cox2*	F	GGTGATGTTTTACATAGTTGGGCGG	218	AB286901	[42]
	R	CCCTTCAAGTTTGGCATTAATCCAC			
*elf*	F	TCGACCACTACAGGTCATCTGATCT	129	LC469958	
	R	CAAGTTATCCAACACCCATGCGT			
*cal*	F	AGGAGCTTGGTACTGTCATGAGATC	104	LC469955	
	R	GTCAATGGTTCCGTTACCATCTGC			
	P	TCAGAATCCAACCGAGGCTGAGT			

**Table 2:** Results of CircWave analysis for diurnal rhythms.

		r^2^ (Rhythmicity)	Acrophase	Amplitude
LD	Fv/Fm ratio	0.95 (<0.01)	16.22	0.01
	O_2_^−^ production	-	23.37	-
	H_2_O_2_ production	-	21.74	-
	H_2_O_2_ scavenging activity	-	15.79	-
	*psbA* expression	0.57 (<0.01)	18.41	0.02
	*psbD* expression	0.77 (<0.01)	15.58	0.01
	*Cu/Zn sod* expression	0.25 (0.015)	16.63	0.69
	*gpx* expression	-	15.68	-
	*cat* expression	0.28 (<0.01)	17.44	0.64
	*apx* expression	0.45 (<0.01)	16.00	0.42
	*trx* expression	-	19.52	-
	*2-Cys prx* expression	0.80 (<0.01)	10.56	7.85
24D	Fv/Fm ratio	0.79 (<0.01)	17.27	0.01
	O_2_^−^ production	-	22.49	-
	H_2_O_2_ production	0.80 (<0.01)	0.67	0.01
	H_2_O_2_ scavenging activity	0.49 (<0.01)	15.43	<0.01
	*psbA* expression	0.53 (<0.01)	21.02	0.01
	*psbD* expression	0.68 (<0.01)	17.15	0.01
	*Cu/Zn sod* expression	0.36 (<0.01)	14.11	0.29
	*gpx* expression	0.25 (0.015)	13.15	0.29
	*cat* expression	0.24 (<0.023)	13.83	1.05
	*apx* expression	0.71 (<0.01)	14.50	0.48
	*trx* expression	-	15.02	-
	*2-Cys prx expression*	0.88 (<0.01)	11.02	10.90

**Table 3:** Results of correlation analysis using Pearson’s correlation coefficients.

		LD		24D	
		*r* Value	*p* Value	*r* Value	*p* Value
*psbA* vs.	Fv/Fm ratio	0.71	<0.01	0.14	0.51
	O_2_^−^ production	0.17	0.59	−0.17	0.41
	H_2_O_2_ production	0.21	0.32	−0.07	0.74
*psbD* vs.	Fv/Fm ratio	0.31	0.15	−0.34	0.10
	O_2_^−^ production	−0.37	0.07	0.12	0.59
	H_2_O_2_ production	0.33	0.12	−0.48	<0.05
*Cu/Zn sod* vs.	Fv/Fm ratio	0.23	0.27	−0.12	0.56
	O_2_^−^ production	−0.21	0.33	−0.24	0.25
	H_2_O_2_ production	0.38	0.07	−0.06	0.78
	H_2_O_2_ scavenging activity	0.26	0.22	0.22	0.29
*gpx* vs.	Fv/Fm ratio	0.01	0.97	−0.27	0.20
	O_2_^−^ production	−0.16	0.45	−0.38	0.07
	H_2_O_2_ production	0.26	0.21	−0.18	0.39
	H_2_O_2_ scavenging activity	0.27	0.19	0.21	0.33
*cat* vs.	Fv/Fm ratio	0.32	0.12	−0.32	0.13
	O_2_^−^ production	−0.07	0.75	−0.24	0.26
	H_2_O_2_ production	0.39	0.06	−0.33	0.12
	H_2_O_2_ scavenging activity	0.20	0.36	0.35	0.09
*apx* vs.	Fv/Fm ratio	0.40	0.05	−0.45	<0.05
	O_2_^−^ production	−0.19	0.37	−0.53	<0.05
	H_2_O_2_ production	0.40	0.05	−0.31	0.13
	H_2_O_2_ scavenging activity	0.33	0.12	0.27	0.20
*trx* vs.	Fv/Fm ratio	0.21	0.33	−0.25	0.24
	O_2_^−^ production	−0.09	0.68	−0.38	0.07
	H_2_O_2_ production	−0.03	0.88	−0.15	0.48
	H_2_O_2_ scavenging activity	0.13	0.55	0.13	0.54
*2-Cys prx* vs.	Fv/Fm ratio	−0.69	<0.01	−0.75	<0.01
	O_2_^−^ production	−0.27	0.21	−0.06	0.76
	H_2_O_2_ production	−0.56	<0.01	−0.63	<0.01
	H_2_O_2_ scavenging activity	−0.25	0.24	−0.35	0.09

## Data Availability

Data are available within the article.
